# Long-Term Residual Infection as a Source of Bovine Tuberculosis Reemergence: A Phylogenetic and Epidemiological Investigation of Recurrent Outbreaks

**DOI:** 10.1155/tbed/2446811

**Published:** 2025-11-07

**Authors:** Bernat Pérez de Val, Mariano Domingo, Alberto Allepuz, Carles Riera, Albert Sanz, Miquel Nofrarías, Sergio López-Soria, Enric Vidal

**Affiliations:** ^1^IRTA, Animal Health Program, Centre for Research in Animal Health (CReSA), Campus of the Autonomous University of Barcelona (UAB), Bellaterra, Cerdanyola del Vallès, Spain; ^2^IRTA-UAB Joint Research Unit in Animal Health, Centre for Research in Animal Health (CReSA), Campus of the Autonomous University of Barcelona (UAB), Bellaterra, Cerdanyola del Vallès, Spain; ^3^WOAH Collaborating Centre for the Research and Control of Emerging and Re-Emerging Swine Diseases in Europe (IRTA-CReSA), Bellaterra, Cerdanyola del Vallès, Spain; ^4^Department of Animal Health and Anatomy, Autonomous University of Barcelona (UAB), Bellaterra, Cerdanyola del Vallès, Spain; ^5^Department of Agriculture, Livestock, Fisheries and Food of the Government of Catalonia, Barcelona, Cerdanyola del Vallès, Spain

**Keywords:** cattle, genomic epidemiology, *Mycobacterium bovis*, residual infection, tuberculosis, whole genome sequencing, wild boar

## Abstract

Bovine tuberculosis (TB), primarily caused by *Mycobacterium bovis*, is a chronic infectious disease of cattle with significant public health and economic implications due to its zoonotic potential and impact on livestock productivity. The control of the disease is hindered by complex epidemiological dynamics and the chronic, and often slow-progressing nature of the disease. The recurrent outbreaks of bovine TB in endemic areas are common and threaten the success of eradication programs. To address this issue, long-term reemergent outbreaks in Catalonia (Northeastern Spain) were retrospectively investigated in depth. In 2009, an outbreak caused by *M. bovis* spoligotype SB0120 was detected in four extensively managed cattle herds. Following intensive eradication measures, all herds recovered the officially TB-free status by 2012. In 2021, 9 years later, a new outbreak involving the same spoligotype was detected in three herds located in the same area, one of which had been affected in the previous outbreak. Extensive sampling of TB-positive slaughtered cattle and hunted wild ungulates was conducted. Whole genome sequencing (WGS) of *M. bovis* isolates from cattle affected in both outbreaks, as well as from two culture-positive wild boars was performed. Epidemiological and phylogenetic analyses were conducted to elucidate the origin and transmission dynamics of the outbreaks. The results revealed a long-term residual infection in the cattle herd that linked the first and second outbreaks. This herd was also the most likely source of transmission of *M. bovis* to wild boars. Since residual infections can jeopardize the final stages of the eradication in low-prevalence settings, thorough investigation of reemerging strains is essential for risk assessment and for guiding TB control decisions.

## 1. Introduction

Mammalian tuberculosis (TB), caused by the *Mycobacterium tuberculosis* complex (MTBC) organisms, is a multihost disease that may affect a range of domestic and wild mammals [1] and can be transmitted to humans [2]. In cattle, TB is predominantly caused by *Mycobacterium bovis* and over 50 million cattle are estimated to be infected worldwide, costing around $ 3 billion every year [3]. The epidemiology of bovine TB involves a complex system, where cattle–to–cattle transmission is considered the primary source of new infections [4]; however, the interface with wildlife, other domestic livestock, and even the environment (e.g., water points and soil) can also contribute to disease incidence [5–7].

While the eradication of bovine TB has been achieved in many European countries, it persists in others despite the huge investment efforts carried out in the last decades. The last stages of disease eradication may be compromised by many factors such as uncontrolled animal movements, shared pastures, interactions with other domestic or wild animals, or residual herd infections [4, 8]. Therefore, identifying the origin of bovine TB outbreaks and those factors influencing the transmission and risk of infection provides valuable insights for developing effective control strategies [9]. Particularly, reemergent outbreaks should be comprehensively investigated to identify the source of reinfection, implement measures to prevent recurrent breakdowns, and mitigate the risk of spreading.

The Spanish bovine TB surveillance is based on three main pillars: routine test–and–culling of reactive cattle to the tuberculin skin test (TST), premovement testing of cattle, and abattoir surveillance for TB-suspicious lesions [10]. Historical TB diagnostic data from each herd are complemented by standardized epidemiological surveys, which are used to support the investigation of the most likely cause of TB outbreaks [9].

In recent years, the use of whole genome sequencing (WGS) of MTBC isolates in TB surveillance has yielded valuable insights into outbreak investigations and disease tracing [11–15]. In this study, we combined WGS data from *M. bovis* isolates with metadata on cattle movements, herd management, and temporal sequence of events to elucidate the transmission dynamics and most likely origin of a recurrent *M. bovis* outbreak affecting both cattle and wild boar.

## 2. Materials and Methods

### 2.1. Eradication Strategy and Epidemiological Data

According to the Spanish bovine TB eradication program [10], all animals older than 6 weeks in cattle herds intended for breeding, meat, milk, or other production purposes, as well as for work, competitions, or exhibitions, shall be subject to at least annual TST. This TB detection protocol is complemented by a slaughterhouse surveillance system for TB-compatible lesions, applied to all animals routinely slaughtered for human consumption. Once a positive herd is detected and confirmed, postmortem by histopathology and PCR or culture, it remains classified as officially TB nonfree until no positive animals are detected in two consecutive tests using TST (single intradermal tuberculin test with severe interpretation: positive reactors when skin fold thickness increase is >2 mm) and interferon-γ release assay (IGRA).

Moreover, in newly positive herds, an epidemiological survey is carried out by a veterinary officer to investigate the cause of the outbreak. A questionnaire registers data related to previous TB testing results, together with epidemiological connections associated with different possible pathways of introduction of the pathogen, such as animal movements (i.e., purchase of animals or movements to pastures), neighboring herds and their TB-status, presence of other domestic animals (e.g., goats), potential interaction points with wildlife reservoirs (e.g., wild boars), and the existence of TB cases among people working in the farm. The opinions of the veterinary officers regarding the most likely cause of the outbreak and their commentaries on additional explanatory factors are also recorded.

### 2.2. Bovine TB Outbreaks History

**Figure 1:**
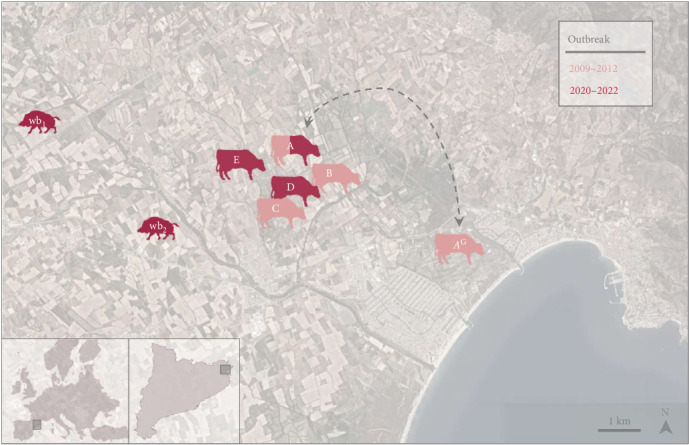
Geolocation of the TB outbreaks. Satellite map showing bovine tuberculosis (TB) outbreaks from two periods: 2009–2012 (light red) and 2020–2022 (dark red). Affected cattle herds (A–E) and wild boar cases (wb_1_–wb_2_) are represented using species-specific silhouettes. AG indicates a grazing area associated with herd A, with a dashed bidirectional arrow denoting the epidemiological link between the two locations. A scale bar indicating 1 km is provided for reference. Inset maps show the position of the outbreak area (Girona, Spain) within Europe and Catalonia.

**Figure 2:**
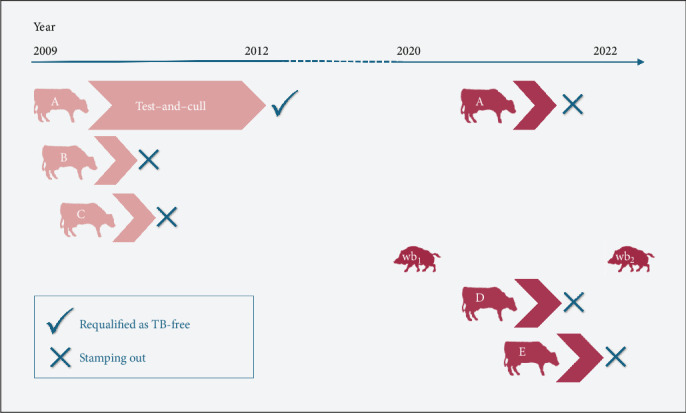
Timeline of *Mycobacterium bovis* detection and eradication events in cattle herds and wild boars. *M. bovis* was detected through slaughterhouse surveillance in cattle herds A (2009 and 2021), B–D, and by tuberculin skin testing (TST) in herd E. Additionally, *M. bovis* was detected in wild boar individuals (wb1 and wb2) from samples collected from hunted animals in 2020 and 2022, respectively. The test–and–cull strategy implemented in herd A between 2009 and 2012 is also indicated. Symbols: “✓” indicates reclassification of the herd as TB-free after test–and–cull; “×” indicates herd stamping out (complete depopulation).

The outbreak area where the involved herds were concentrated (Figure 1) is an agricultural zone located between the inland and the coast of the province of Girona (Catalonia, Spain). The timeline of *M. bovis* detection and eradication actions is outlined in Figure 2.

### 2.3. Outbreak 1 (2009–2012)

In June 2009, a bovine with TB-compatible lesions was detected through routine slaughterhouse inspection. The following month, another bovine from a different herd exhibited similar lesions at the same abattoir. In both cases, TB was confirmed by postmortem examination, followed by *M. bovis* isolation and identification. The animals belonged to two beef cattle herds under extensive management within the same municipality in Girona province (Figure 1).

The first herd was divided into two locations, separated by 5.4 km: the main farm (A), which housed 88 animals, including the confirmed TB case and a grazing area (*A*^G^) with 96 animals. The second herd (B), with 96 animals, was located just 800 m from herd A. Both herds were included in an intensive test-and-slaughter eradication plan, with animals undergoing periodic testing with TST and IGRA every 2–3 months. This strategy led to herd A and *A*^G^ achieving TB-free status by December 2012 and October 2011, respectively. On the other hand, the high proportion of positive animals (63%) in the initial testing conducted in herd B prompted authorities to conduct the herd stamping out in August 2009.

Additionally, in November 2009, a third case of TB was detected through slaughterhouse inspection in a fattening calf herd (C), which was located less than 500 and 1200 m apart from herds B and A, respectively. Since the whole batch of fattening calves from herd C was slaughtered and the herd was completely depopulated at that time point, no further action was required for this herd.

### 2.4. Outbreak 2 (2020–2022)

Eleven years after Outbreak 1, in November 2020, TB infection was confirmed in a hunted wild boar located about 5.5 km from herds A and B. In April 2021, five new animals from herd A showed TB-compatible lesions during a routine abattoir inspection, which were later laboratory confirmed as TB due to *M. bovis* by culture. A total of 40 out of 77 animals tested positive for the TST and/or IGRA the following month. Additionally, in the same month, a 13-year-old bovine from another beef cattle herd (D), located at <1 km from either A or B was detected with generalized TB also through slaughterhouse inspection. All cattle herds within a 5 km radius from herd A were subsequently tested, and in August of the same year, the TST revealed four positive cattle of a dairy herd (E) that were confirmed as TB-infected. Stamping out was carried out in all three affected herds (A, D, and E) during 2021. Sampling of hunted wild boars was intensified in this area, and in November 2022, a second wild boar with TB was detected less than 3 km from the previously affected herds.

### 2.5. Sampling

The tissue samples from cattle were obtained in the framework of the Spanish bovine TB eradication program from slaughtered TST reactors or TB-suspicious lesions at abattoirs. All samples were submitted through the slaughterhouse support network ([16], www.sesc.cat) to the laboratory at IRTA-CReSA (Bellaterra, Barcelona). Additionally, wild boar tissues were obtained from hunted animals in the framework of the Catalan wildlife health surveillance plan, initiated in 2014. A total of 85 wild boars were sampled in the outbreak area between 2020 and 2022.

### 2.6. Bacterial Isolation and Identification

Mycobacterial isolation from tissue samples was performed as previously described [17] in Löwenstein–Jensen with Pyruvate or Coletsos solid media (BD Diagnostics, Sparks, MD, USA) or by the BACTEC MGIT system (BD Diagnostics). MTBC was identified by multiplex PCR [18]. Fifteen MTBC-confirmed isolates were selected for WGS analysis. These included five isolates from the 2009–2012 outbreak, nine from the 2020–2022 outbreak (with at least one sample per herd and outbreak as well as two wild boar samples), and one additional isolate from cattle in a neighboring county in 2009, used as an outgroup. These isolates were subcultured on 7H11 plates (BD Diagnostics) at 37°C for 28 days. Bacterial growths were suspended in 1 mL PBS and inactivated at 100°C for 30 min. Inactivated samples were sent to the National Veterinary Services Laboratories (NVLS) of the United States Department of Agriculture (USDA, Ames, IA) for WGS.

### 2.7. WGS and Data Analysis

Genomic DNA from the 15 isolates (14 from the outbreaks and one outgroup) was extracted at the NVLS of the USDA (Ames, IA, United States) as previously described [19]. WGS was performed using Illumina Miseq (Illumina, Inc., San Diego, CA, USA). All sequences were aligned to the reference genome *M. bovis* AF2122/97 (NCBI Accession Number NC_002945.4). The sequences are available at the GenBank (Bioproject Number PRJNA384785).

Single nucleotide polymorphisms (SNPs) were identified using the vSNP pipeline (https://github.com/USDA-VS/vSNP). Phylogenies were constructed using Randomized Axelerated Maximum Likelihood (RAxML) following the procedures described previously [20]. DVR–spoligotyping profiles were also determined in silico through the vSNP pipeline. The “spoligo” function generated a binary code as result of the read counts for each of the oligonucleotide spacer regions. This code was cross-referenced with the *M. bovis* spoligotype database (www.mbovis.org).

An intermediate pairwise SNP distance threshold of ≤ 8 SNPs was defined to provide sufficient resolution to identify transmission clusters within the outbreak while avoiding the exclusion of older transmission events. This criterion was based on the previously described threshold range of 5–12 SNPs for clustering both recent and older linked cases [21, 22].

The Microreact [23] and PHYLOViZ [24] web-based platforms were used for visualizing, annotating, and editing the phylogenetic trees and to construct the minimum spanning tree (MST), respectively.

## 3. Results

### 3.1. Epidemiological Investigation

### 3.2. Outbreak 1

Herds A and B were detected simultaneously at the slaughterhouse, each with one animal with TB suspected lesions in lungs and pulmonary lymph nodes that were subsequently confirmed by PCR and/or culture. Although it was uncertain which of the two was the primary case, herd B was the most likely source of infection, given the higher number of positive animals in the initial testing with TST and IGRA carried out after detection at the slaughterhouse (60 out of 96 in herd B vs., 26 out of 99 in herd A).

In contrast, in the grazing area associated with herd A (*A*^G^), located 5.4 km from the main farm facilities, only one out of 67 cattle (1.5%) tested positive and was confirmed as TB-infected after slaughter. The epidemiological survey recorded no recent animal movements between *A* and *A*^G^ (the last recorded movement was in April 2008, 15 months before the outbreak was detected). However, both herds were managed as a single epidemiological unit, since all animals in *A*^G^ originally came from *A*. Similarly, only one case of TB was confirmed in farm C (out of 160 slaughtered animals). The epidemiological investigation recorded animal movements from *B* to *C* for fattening purposes.

No direct contact between animals from farms A and B was recorded, despite their proximity (within a radius of around 800 m). Nevertheless, the records noted that such contacts were highly likely, given that both farms operated under extensive management systems and had limited biosecurity measures.

### 3.3. Outbreak 2

The index case of this second outbreak was a wild boar (wb_1_) with TB lesions detected in November 2020. Records indicated that the three cattle herds detected in 2021 (A, D, and E) had a history of negative TST results in previous years (annual testing). However, herd A, only 1 month after the detection of five animals with TB lesions at slaughterhouse, showed a high rate of positivity in the initial testing with 44 out of 77 animals to TST or IGRA.

Herd D had only one TB case, a 13-year-old animal detected at the slaughterhouse with generalized TB (pearl disease form), while none of the other animals in the herd (54 in total) showed TB lesions nor positive culture. The epidemiological survey highlighted that herds A and D were managed extensively and were separated only by an electric fence but allowing the possibility of direct contact between them. In addition, they may also have shared water sources (drinking points for the animals). Herd D was also very close to herds B and C (>400 m away), but no evidence of direct contact or shared spaces was reported.

Herd E was investigated ad hoc immediately after the TB cases in herds A and D were detected, due to its proximity (<0.6 km, Figure 1). It showed four out of 238 positive animals to the TST (3 of which were confirmed as TB-infected). The epidemiological investigation reported no direct contact with other herds, but the farm was not fenced, and the epidemiological survey mentioned the possibility of contact with wildlife or, more remotely, with cattle from other herds that occasionally “escaped.”

Finally, in 2022, a second wild boar with TB (wb_2_) was detected. Thus, two out of the 85 (2%) wild boars sampled in the area between 2020 and 2022 were confirmed as TB-infected.

### 3.4. Phylogeny of the Outbreaks

*M. bovis* with the spoligotype profile SB0120 was identified in silico in the 14 sequences from the outbreaks (12 from cattle and 2 from wild boars) as well as in the outgroup sequence.

**Figure 3:**
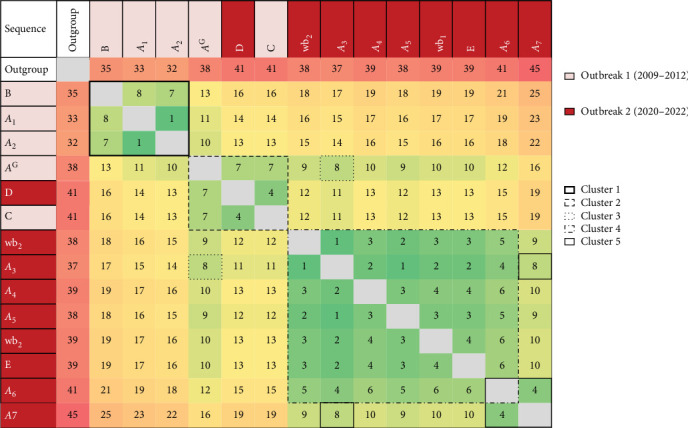
Pairwise SNP distance matrix among *Mycobacterium bovis* isolates. The matrix displays the number of SNP differences between all isolates included in the study: Outbreak 1 (*N* = 5, light red), Outbreak 2 (*N* = 9, dark red), and an outgroup sequence (white). SNP distances were computed using maximum-likelihood phylogenetic inference (RAxML) and visualized as a symmetric matrix. A clustering threshold of ≤ 8 SNPs was applied to define genetic clusters (Clusters 1–5), delineated by colored dashed boxes. Cell color gradients represent increasing genetic distances: green ≤ 8 SNPs (within-cluster distances), yellow: 9–15 SNPs (moderate proximity), orange: 16–25 SNPs (moderate divergence, still within the outbreaks), and red: >25 SNPs (high divergence, outgroup). Isolates from cattle herds (A to E) or wild boars (wb1–wb2) are indicated.

All SNPs identified are shown in Supporting Information 1: S1. The maximum pairwise difference among all sequences from the two outbreaks was 25 SNPs (16 SNPs in Outbreak 1 and 19 SNPs in Outbreak 2; Figure 3), while the maximum difference between most recent common ancestors (MRCAs) was 20 SNPs (11 SNPs in Outbreak 1 and 14 SNPs in Outbreak 2; Supporting Information 2: S2).

**Figure 4:**
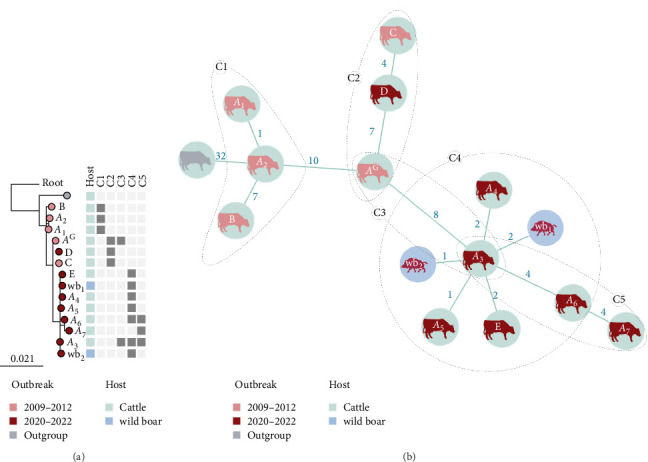
Phylogenetic trees based on whole genome sequences of *Mycobacterium bovis* isolates. (A) Maximum-likelihood (RAxML) phylogenetic tree including 15 isolates from 2009 to 2012 outbreak (light red circles), the 2020–2022 outbreak (dark red circles), and the outgroup sequence (gray circle). Host types (cattle or wild boar) and the five clusters (C1–C5; threshold for clustering: ≤ 8 SNP) are represented according to the legend. Cattle herd (A–E) or wild boars (wb1–wb2) are also indicated. Root: *M. bovis* AF2122/97 reference strain sequence (National Center for Biotechnology Information Accession Number NC_0002945). Scale: Number of nucleotide substitutions per site. (B) Minimum-spanning tree (MST) of the most likely transmission path within and between the outbreaks. Each node (circle) represents a sequence (isolate). Numbers show the SNP pairwise distance between sequences. Silhouettes inside each circle represent the host (cattle or wild boar). Cattle herds or wild boars are identified in the silhouette. Clusters grouping sequences are also indicated and delineated by dashed lines.

To infer transmission clusters, including old and more recent sequences, a pairwise SNP distance threshold of ≤ 8 SNPs was used. Five distinct clonal clusters were identified (Figures 3 and 4A). Cluster 1 (C1) comprised sequences from herds A and B, all associated with Outbreak 1. Cluster 2 (C2) included sequences from the pasture *A*^G^ and herd C (both from Outbreak 1), as well as the isolate from herd D (Outbreak 2). The isolate from *A*^G^ (2009) and the isolate *A*_3_ (herd A, 2021) constituted Cluster 3 (C3). Cluster 4 (C4) encompassed sequences from herds A and E, along with the two wild boars, all from Outbreak 2. Finally, Cluster 5 (C5) was formed by three sequences from herd A, also from Outbreak 2.

The most likely evolutionary paths and putative transmission clusters were assessed using a MST, which connects all sequences with the shortest possible total SNP distance (Figure 4B). The *A*^G^ isolate (Outbreak 1) was identified as the most probable ancestor node shared by both C2 and C3 clusters (the latter also putative precursor of C4 and C5 clusters). The *A*^G^ isolate sequence showed 7-SNP pairwise distance with the other two C2 sequences (5-SNP from their common ancestor) and 8-SNP distance (6-SNP from their common ancestor) with the other C3 sequence, the *A*_3_ isolate, belonging to the Outbreak 2 (Figure 3 and Supporting Information 2: S2).

According to MST, the isolates from herd E and the two wild boars evolved from the *A*_3_ isolate (herd A). Interestingly, all the isolates of the Outbreak 2 were grouped in C4 and the subsequent C5 (that evolved from C4), except the isolate from herd D (the 13-year-old bovine). The latter belonged to C2, an independent branch also involving the herd C isolate (Outbreak 1) and the *A*^G^ isolate (Figure 4B).

## 4. Discussion

By combining epidemiological and genomic data, this study revealed recurrent *M. bovis* infections across two temporally separated outbreak periods, with residual infection in a single cattle herd (i.e., the continuous presence of animals with undetected infection) as the most probable source of reemergence.

Although the exact origin of the outbreak detected in 2009 (Outbreak 1) cannot be determined with certainty, it is likely that *M. bovis* had been circulating within and between herd A and herd B for several years prior to outbreak detection. This is supported by the high number of animals testing positive in the initial screening (26/99 and 60/96, respectively), which implies several years of infection based on the transmission rates described for bovine TB. Specifically, in Spain, Ciaravino et al. [25] estimated a median transmission coefficient (β) of 5.2 newly infected cattle per infectious individual per year.

In contrast, although the Outbreak 2 was initially detected in a wild boar in 2020 (the first case that enabled the identification of the outbreak), retrospective genomic epidemiology analysis clearly revealed that the primary case of this outbreak was in herd A, detected several weeks later in 2021. In this regard, according to transmission inference using MST, the two detected wild boars and the isolate from herd E differ by one or two SNPs derived from a common ancestor identified in an isolate from herd A (isolate *A*_3_). The sequence of this isolate represented a genetic bottleneck explaining all the transmission paths within Outbreak 2, except from the herd D isolate, which is discussed later in this section.

As in Outbreak 1, herd A exhibited a high rate of positivity (57%) in the initial testing conducted 1 month after abattoir detection of five animals with TB lesions of this herd in Outbreak 2. In addition, a pairwise SNP distance of up to 10 SNPs was observed among isolates from this herd, indicating a longstanding, undetected infection and extensive intraherd transmission. Notably, herd A had already been involved in Outbreak 1, and the epidemiological investigation revealed that one of the TB-positive animals was the offspring of a cow that tested positive in 2009. These findings collectively suggest a residual infection within the herd, likely representing the source of *M. bovis* SB0120 reemergence in the area. The detection of both outbreaks through slaughterhouse surveillance underscored limitations in the effectiveness of the annual TST in these herds.

One of the limitations of this study is that not all infected animals were submitted for culture and subsequent WGS, resulting in missing isolate sequences that hinder the full reconstruction of transmission chains. Therefore, since all animals of the grazing area *A*^G^ originally came from herd A, the single animal confirmed as TB-infected in *A*^G^ was likely infected by another unsampled animal from herd A carrying a *M. bovis* strain with an identical genome sequence. This isolate recovered from *A*^G^ in 2009 is particularly relevant, as phylogenetic analysis revealed a close relationship with an isolate from herd A in 2021 (*A*_3_), which was identified as the common ancestor of most sequences from Outbreak 2. These two sequences were separated by only 8 SNPs, with a maximum of 5 SNPs from their inferred common ancestor. Considering the 12-year period between isolations, this corresponds to a substitution rate of approximately 0.4 SNPs/year, based on the time elapsed since the common ancestor. This rate falls within the range of 0.015–0.5 SNPs/year reported for other *M. bovis* outbreaks [26, 27] and supports the hypothesis of a residual infection within the herd.

Intriguingly, the 2021 isolate from herd D was positioned phylogenetically on a branch independent from the rest of the isolates collected between 2020 and 2022, clustering instead with an isolate from herd C and the isolate *A*^G^, both dated 2009. However, there were epidemiological links between herd A and herd D, as both herds were separated only by a fence and probably shared water sources. The fact that the animal from herd D was already present in the herd in 2009 (being 13-years-old at the time of detection) suggests that it may have been infected during Outbreak 1, and that the infection remained undetected throughout the inter-outbreak period. Even though *M. bovis* primarily induces a progressive active infection, latent infection cannot be ruled out in ruminants, since immune mechanisms underlying latency are not well characterized yet [28]. Thus, a decline in host immunity associated with advanced age could have led to reactivation of the infection, potentially resulting in a fulminant disease progression and induction of generalized lesions [29], as those observed in this animal at slaughterhouse. This case highlights the need to accelerate the replacement of older animals, regardless of negative TST results, particularly in areas with a high risk of TB exposure. Moreover, the detection of this case, concurrent but likely independent from the other cases, highlights two distinct aspects of residual herd infection: misdiagnosis and immunological unresponsiveness, representing different ways through which infection may persist undetected within a herd.

In this regard, Wiseman and collaborators [30] described the residual infection as an “umbrella,” including both infected nonreactive animals and infected misdiagnosed ones. The first group mainly includes animals in early or latent stages of the infection, as well as immunosuppressed or *M. bovis* tuberculin-desensitized animals [31], while the second implies a failure in the diagnosis due to several reasons, such as a lack of alignment between the timing of testing and TB transmission rate, incomplete testing of the entire herd, or the intrinsic limitations of the diagnostic tests, particularly TST [32]. In this regard, the inherent subjectivity of the TST may have contributed to misdiagnosis in this study, particularly during the first outbreak in 2009, when veterinarians performing field tests were not yet subjected to reinforced monitoring tools. Subsequently, starting in 2013, theoretical and practical training courses were implemented for all veterinarians to enable them to carry out the TST, alongside a strengthened official control plan to supervise the field veterinarians conducting the tests [10].

The role of residual infection in the epidemiology of bovine TB is particularly relevant in low-prevalence bovine TB scenarios, when the decline in the positive predictive value of diagnostic tests may lead to TST reactions being interpreted as false positives or to the misclassification of lesions as non-TB granulomas during slaughterhouse inspections, ultimately resulting in a higher proportion of infected animals remaining undetected [33]. In Spain, different studies found residual infection as the most likely source of newly bovine TB cases together with the cattle–wildlife interactions [4, 9].

Finally, the sequence from herd E is phylogenetically derived from sequence *A*_3_ (2 SNPs), and the fact that the epidemiological survey reported contacts with escaped animals from herd A suggests that this neighboring interaction is the most likely source of infection for herd E. Although indirect interaction with an infected wild boar cannot be completely ruled out, the epidemiological survey reported no contacts between the herd and wildlife. Furthermore, among the 85 wild boars analyzed between 2020 and 2022 in this area, only two were confirmed as TB-infected, and these two were not directly related to each other (their isolates were linked through sequence *A*_3_), and both sequences are evolutionarily derived from a common ancestor isolated in cattle (see Figure 4). While the presence of undetected infected wild boars cannot be excluded, there is no evidence in this study of *M. bovis* maintenance within the wild boar population, nor of transmission from wild boars to cattle. Moreover, no cases of TB were detected in other wildlife species sampled in the framework of wildlife health surveillance plan nor in nonbovine livestock in the outbreak area, the latter also subjected to slaughterhouse surveillance.

Reinfections may jeopardize the final stages of bovine TB eradication campaigns. In addition to external sources of infection, such as transmission from other domestic or wild reservoirs or exposure to contaminated environments in certain epidemiological conditions, the persistent presence of infected but undetected animals within herds represents a significant risk of bovine TB recurrence. All these cases not only pose a risk to epidemiologically related TB-free herds but also have an enormous impact in terms of economic losses and farmers confidence in eradication strategies [34].

## 5. Conclusions

In this study, we investigated recurrent bovine TB outbreaks involving hidden *M. bovis* transmission, which affected a total of five cattle herds and local wildlife. All five herds were subjected to stamping out at some point during the outbreak management process. Results obtained through the analysis of WGS data together with epidemiological data, from affected herds suggest that during a long period (over 8 years) there was an undetected residual infection and hidden transmission in the area. The findings underscore the importance of thorough epidemiological investigations in recurrent TB cases, to understand the emergence of new cases in previously affected areas and to identify weaknesses that enable both undetected residual infections and silent transmission among epidemiologically related herds, as well as between livestock and wildlife.

In this context, integrating genome surveillance data from high-throughput sequencing technologies has proven valuable for elucidating transmission dynamics and conducting accurate risk assessments [12, 22, 35, 36]. Our findings also support the adoption of risk-based surveillance strategies to implement stricter control measures in areas with a high risk of TB recurrence due to residual infection. These may include increased testing frequency [37], accelerated replacement of older animals, or herd stamping out once infection is established, along with reinforcement of biosecurity measures and wildlife population control, to minimize the risk of recurrence.

## Data Availability

The data that support the findings of this study are available from the corresponding author upon reasonable request.

## Supporting Information

Supporting Information 1 Supporting Information S1: List of SNPs identified among *Mycobacterium bovis* isolates.

Supporting Information 2 Supporting Information S2: Pairwise SNP distance matrix from the most recent common ancestor (MRCA) among *Mycobacterium bovis* isolates.

## References

[1] WOAH, World Organisation for Animal Health (WOAH) (2023). Mammalian Tuberculosis (infection With *Mycobacterium tuberculosis* Complex). *Manual of Diagnostic Tests and Vaccines for Terrestrial Animals*.

[2] Müller B., Dürr S., Alonso S. (2013). Zoonotic *Mycobacterium bovis*-induced Tuberculosis in Humans. *Emerging Infectious Diseases*.

[3] Waters W. R., Palmer M. V., Buddle B. M., Vordermeier H. M. (2012). Bovine Tuberculosis Vaccine Research: Historical Perspectives and Recent Advances. *Vaccine*.

[4] Guta S., Casal J., Napp S. (2014). Epidemiological Investigation of Bovine Tuberculosis Herd Breakdowns in Spain 2009/2011. *PLoS ONE*.

[5] Naranjo V., Gortazar C., Vicente J., de la Fuente J. (2008). Evidence of the Role of European Wild Boar as a Reservoir of *Mycobacterium tuberculosis* Complex. *Veterinary Microbiology*.

[6] Napp S., Allepuz A., Mercader I. (2013). Evidence of Goats Acting as Domestic Reservoirs of Bovine Tuberculosis. *Veterinary Record*.

[7] Barasona J. A., Vicente J., Díez-Delgado I., Aznar J., Gortázar C., Torres M. J. (2017). Environmental Presence of *Mycobacterium tuberculosis* Complex in Aggregation Points at the Wildlife/Livestock Interface. *Transboundary and Emerging Diseases*.

[8] More S. J., Good M. (2015). Understanding and Managing bTB Risk: Perspectives From Ireland. *Veterinary Microbiology*.

[9] Ciaravino G., Laranjo-González M., Casal J., Sáez-Llorente J. L., Allepuz A. (2021). Most Likely Causes of Infection and Risk Factors for Tuberculosis in Spanish Cattle Herds. *Veterinary Record*.

[10] MAPA (2024). Programa Nacional De Erradicación De Tuberculosis Bovina 2025 (Infección por el Complejo *Mycobacterium tuberculosis*). https://www.mapa.gob.es/dam/mapa/contenido/ganaderia/temas/sanidad-animal-e-higiene-ganadera/sanidad-animal/enfermedades/bovino/tuberculosis-bovina/programas-nacionales/programatb2025_18022025.pdf.

[11] Akhmetova A., Guerrero J., McAdam P. (2023). Genomic Epidemiology of *Mycobacterium bovis* Infection in Sympatric Badger and Cattle Populations in Northern Ireland. *Microbial Genomics*.

[12] Price-Carter M., Brauning R., de Lisle G. W. (2018). Whole Genome Sequencing for Determining the Source of *Mycobacterium bovis* Infections in Livestock Herds and Wildlife in New Zealand. *Frontiers in Veterinary Science*.

[13] Rossi G., Crispell J., Brough T. (2022). Phylodynamic Analysis of an Emergent *Mycobacterium bovis* Outbreak in an Area With no Previously Known Wildlife Infections. *Journal of Applied Ecology*.

[14] Ciaravino G., Vidal E., Cortey M. (2021). Phylogenetic Relationships Investigation of *Mycobacterium caprae* Strains From Sympatric Wild Boar and Goats Based on Whole Genome Sequencing. *Transboundary and Emerging Diseases*.

[15] Pereira A. C., Reis A. C., Cunha M. V. (2023). Genomic Epidemiology Sheds Light on the Emergence and Spread of *Mycobacterium bovis* Eu2 Clonal Complex in Portugal. *Emerging Microbes & Infections*.

[16] Vidal E., Tolosa E., Espinar S. (2016). 6-Year Follow-up of Slaughterhouse Surveillance (2008–2013): The Catalan Slaughterhouse Support Network (SESC).

[17] Melgarejo C., Cobos A., Domingo M. (2024). Experimental Infection of Goats With *Mycobacterium microti* Induces Subclinical Pulmonary Tuberculosis and Mild Responses to Tuberculin Skin Tests. *Veterinary Microbiology*.

[18] Wilton S., Cousins D. (1992). Detection and Identification of Multiple Mycobacterial Pathogens by DNA Amplification in a Single Tube. *Genome Research*.

[19] Perea C., Ciaravino G., Stuber T. (2021). Whole-Genome SNP Analysis Identifies Putative *Mycobacterium bovis* Transmission Clusters in Livestock and Wildlife in Catalonia, Spain. *Microorganisms*.

[20] Hicks J., Stuber T., Lantz K., Torchetti M., Robbe-Austerman S. (2024). VSNP: A SNP Pipeline for the Generation of Transparent SNP Matrices and Phylogenetic Trees From Whole Genome Sequencing Data Sets. *BMC Genomics*.

[21] Nikolayevskyy V., Kranzer K., Niemann S., Drobniewski F. (2016). Whole Genome Sequencing of *Mycobacterium tuberculosis* for Detection of Recent Transmission and Tracing Outbreaks: A Systematic Review. *Tuberculosis*.

[22] Walker T. M., Ip C. L., Harrell R. H. (2013). Whole-Genome Sequencing to Delineate *Mycobacterium tuberculosis* Outbreaks: A Retrospective Observational Study. *The Lancet Infectious Diseases*.

[23] Argimón S., Abudahab K., Goater R. J. E. (2016). Microreact: Visualizing and Sharing Data for Genomic Epidemiology and Phylogeography. *Microbial Genomics*.

[24] Francisco A. P., Vaz C., Monteiro P. T., Melo-Cristino J., Ramirez M., Carriço J. A. (2012). PHYLOViZ: Phylogenetic Inference and Data Visualization for Sequence Based Typing Methods. *BMC Bioinformatics*.

[25] Ciaravino G., García-Saenz A., Cabras S. (2018). Assessing the Variability in Transmission of Bovine Tuberculosis Within Spanish Cattle Herds. *Epidemics*.

[26] Trewby H., Wright D., Breadon E. L. (2016). Use of Bacterial Whole-Genome Sequencing to Investigate Local Persistence and Spread in Bovine Tuberculosis. *Epidemics*.

[27] Crispell J., Zadoks R. N., Harris S. R. (2017). Using Whole Genome Sequencing to Investigate Transmission in a Multi-Host System: Bovine Tuberculosis in New Zealand. *BMC Genomics*.

[28] García J. S. Y., Bigi M. M., Klepp L. I., García E. A., Blanco F. C., Bigi F. (2020). Does *Mycobacterium bovis* Persist in Cattle in a Non-Replicative Latent State as *Mycobacterium tuberculosis* in Human Beings?. *Veterinary Microbiology*.

[29] Domingo M., Vidal E., Marco A. (2014). Pathology of Bovine Tuberculosis. *Research in Veterinary Science*.

[30] Wiseman J., Cassidy J. P., Gormley E. (2024). The Problem That Residual *Mycobacterium bovis* Infection Poses for the Eradication of Bovine Tuberculosis. *The Veterinary Journal*.

[31] de la Rua-Domenech R., Goodchild A. T., Vordermeier H. M., Hewinson R. G., Christiansen K. H., Clifton-Hadley R. S. (2006). Ante Mortem Diagnosis of Tuberculosis in Cattle: A Review of the Tuberculin Tests, Y-Interferon Assay and Other Ancillary Diagnostic Techniques. *Research in Veterinary Science*.

[32] Conlan A. J. K., McKinley T. J., Karolemeas K. (2012). Estimating the Hidden Burden of Bovine Tuberculosis in Great Britain. *PLoS Computational Biology*.

[33] Radunz B. (2006). Surveillance and Risk Management During the Latter Stages of Eradication: Experiences From Australia. *Veterinary Microbiology*.

[34] Ciaravino G., Espluga J., Casal J., Pacios A., Mercader I., Allepuz A. (2020). Profiles of Opinions Among Farmers and Veterinarians Towards the Tuberculosis Eradication Programme in Cattle in Spain. *Preventive Veterinary Medicine*.

[35] Allen A., Magee R., Devaney R. (2024). Whole-Genome Sequencing in Routine *Mycobacterium bovis* Epidemiology — Scoping the Potential. *Microbial Genomics*.

[36] de Val B. P., Vidal E., Stuber T., Sáez J. L., Tórtola M. T. (2025). Zoonotic Tuberculosis in Catalonia, Spain: Phylogenetic Insights Into *Mycobacterium bovis* and *M. caprae* Transmission at the Human-Livestock Interface. *One Health*.

[37] Napp S., Ciaravino G., de Val B. P., Casal J., Saéz J. L., Alba A. (2019). Evaluation of the Effectiveness of the Surveillance System for Tuberculosis in Cattle in Spain. *Preventive Veterinary Medicine*.

